# Current In Vitro Models to Study Varicella Zoster Virus Latency and Reactivation

**DOI:** 10.3390/v11020103

**Published:** 2019-01-26

**Authors:** Nicholas L. Baird, Shuyong Zhu, Catherine M. Pearce, Abel Viejo-Borbolla

**Affiliations:** 1 Department of Neurology, University of Colorado School of Medicine, Aurora, CO 80045, USA; Catherine.Pearce@ucdenver.edu; 2 Institute of Virology, Hannover Medical School, 30625 Hannover, Germany; Zhu.Shuyong@mh-hannover.de

**Keywords:** varicella zoster virus, stem cells, human neurons, latency, reactivation, neurotrophic factors

## Abstract

Varicella zoster virus (VZV) is a highly prevalent human pathogen that causes varicella (chicken pox) during primary infection and establishes latency in peripheral neurons. Symptomatic reactivation often presents as zoster (shingles), but it has also been linked to life-threatening diseases such as encephalitis, vasculopathy and meningitis. Zoster may be followed by postherpetic neuralgia, neuropathic pain lasting after resolution of the rash. The mechanisms of varicella zoster virus (VZV) latency and reactivation are not well characterized. This is in part due to the human-specific nature of VZV that precludes the use of most animal and animal-derived neuronal models. Recently, in vitro models of VZV latency and reactivation using human neurons derived from stem cells have been established facilitating an understanding of the mechanisms leading to VZV latency and reactivation. From the models, c-Jun N-terminal kinase (JNK), phosphoinositide 3-kinase (PI3K) and nerve growth factor (NGF) have all been implicated as potential modulators of VZV latency/reactivation. Additionally, it was shown that the vaccine-strain of VZV is impaired for reactivation. These models may also aid in the generation of prophylactic and therapeutic strategies to treat VZV-associated pathologies. This review summarizes and analyzes the current human neuronal models used to study VZV latency and reactivation, and provides some strategies for their improvement.

## 1. Introduction

Varicella zoster virus (VZV) is a human neurotropic virus that establishes latency in sensory and autonomic neurons of the peripheral nervous system (PNS). VZV DNA has been detected in neurons of sympathetic, enteric, trigeminal and dorsal root ganglia (TG and DRG, respectively) [1,2]. Clinical and basic research evidence suggests VZV can reactivate from any of these neurons; however, infectious VZV has never been recovered from any explanted ganglion [3,4,5,6,7,8]. Primary VZV infection causes varicella (chickenpox). VZV transcription and translation profile during latency is controversial, with contradicting reports regarding the level of VZV gene expression. However, available data indicate that VZV latency in vivo is characterized by minimal gene expression (one or two genes), lack of detectable mRNA translation and no virus production [9,10]. Upon symptomatic reactivation, VZV can cause zoster (shingles), which is characterized by a painful rash in the dermatome innervated by the ganglion where reactivation occurred. Shingles may be followed by postherpetic neuralgia (PHN), the second most common type of neuropathic pain worldwide. VZV reactivation can also cause other pathologies such as zoster sine herpete, vasculopathy, meningoencephalitis, encephalitis, etc. ([11], reviewed by Peter Kennedy and Anne Gerson in this Special Issue [12]). Importantly, VZV DNA and infectious virus have been found in saliva of individuals without clinical symptoms of VZV reactivation, demonstrating asymptomatic VZV reactivation [13,14,15]. Currently, two vaccines to reduce VZV reactivation are available—Zostavax (live, attenuated; Merck) and Shingrix (subunit; GlaxoSmithKline). However, the use of the licensed vaccines is not universal [16] and therefore a large percentage of the global population is not vaccinated against VZV. Furthermore, neither vaccine is 100% effective at preventing VZV reactivation. An understanding of the mechanisms leading to VZV latency and reactivation may facilitate the generation of novel therapeutics against VZV-induced disease, as well as improve current vaccines aimed at protecting against zoster and PHN.

Several in vivo models have been developed to study VZV neurotropism, latency, and reactivation. Despite being informative, these in vivo models are not suitable for mechanistic studies. Moreover, they are not available to the wide scientific community because they are technically demanding, costly and it is not possible to obtain ethical approval for some of these experiments in certain countries. Technological advances have facilitated the generation of different neuronal subtypes, which have been used to study VZV biology. Stem cell-derived human neurons allow for mechanistic studies to investigate VZV latency and reactivation, modulation of neuronal activity by VZV and whether VZV infection proceeds equally in different neuronal subtypes. Here, we review the current literature on VZV latency and reactivation obtained with human neurons, focusing on the currently available in vitro neuronal models. The use of these models has already shed light on critical aspects of VZV latency and reactivation and provides an opportunity to address unresolved questions. However, these are exactly that—models—attempting to recreate a state that only truly exists in vivo, where an intact immune system, support cells (e.g., Schwann cells), and a 3D matrix exists. The immune system is critical in the latency/reactivation balance—its loss or suppression often leads to VZV reactivation [17,18,19,20,21,22]. As is reviewed in the “Concluding remarks and outlook” section, these models are very informative, but inherently all current models fall short of what presumably occurs in vivo.

## 2. Characteristics of VZV Latency

The human alphaherpesviruses VZV, herpes simplex virus type 1 (HSV-1) and 2 (HSV-2), exist in one of two phases: lytic (productive) or latent (silent). Most of the knowledge regarding alphaherpesvirus latency has been obtained using HSV-1 in animal or in vitro models using murine neurons. Importantly, the conclusions obtained with HSV-1 may not apply to VZV. For a more detailed review on molecular aspects of VZV latency, the reader is referred to another review in this Special Issue [10]. The generation of in vitro models to study VZV latency and reactivation will provide answers to many unsolved questions on this topic.

During primary infection (lytic phase) of a cell, there is an ordered cascade of gene expression starting with immediate early (IE) followed by early (E) and late (L) genes. Transcription of IE genes is promoted by the interaction of certain tegument proteins (e.g., VZV ORF10 protein or HSV-1 VP16) with cellular proteins, including host cell factor 1 (HCF-1). This results in the acquisition of active chromatin marks in the promoters of IE genes, facilitating their expression [23,24]. During infection of neurons at their termini, ORF10 and VP16 are released from incoming virions and transported to the nucleus independently from the capsid, travelling long distances, separated from the capsid, and thus are inefficiently delivered to the nucleus. Moreover, HCF-1 localizes in the cytoplasm of neurons, contrary to its nuclear localization in other cell types [25]. This, together with the possible presence of repressors of gene expression, may contribute to transcriptional silencing of the viral genome, leading to viral latency [24,26]. Indeed, VZV genomes are subjected to epigenetic modifications in human TG neurons [27].

Alphaherpesvirus latency is characterized by episomal maintenance of the viral genome and a state of chronic infection that does not result in production of infectious viral particles [28]. This state is not abortive since reactivation, and therefore the generation of infectious virus, can occur. Normally, viral gene expression is highly restricted during latency and constrained to that of one or a few genes. These are the dogmatic views of what is believed to occur in vivo, based on the current data. However, there is almost assuredly a mixture of neuron/virus interaction states—some virus genomes may never reactivate and some genomes may not express any genes whereas others may express several. Initial studies of human ganglia obtained at autopsy (less than 24 hours following death) detected transcription and translation of several VZV open reading frames (ORFs) [29,30,31,32,33]. This differed from latency of the other two human alphaherpesviruses, HSV-1 and -2, where a more restricted gene expression profile is observed. Each HSV expresses a non-coding RNA termed “latency associated transcript” (LAT), micro RNAs (miRNAs) and small RNAs; though, no protein has been convincingly detected during HSV latency (reviewed in [34]). Therefore, it seemed that VZV had a more relaxed transcription and translation pattern than HSV during latency. However, advances in research are demonstrating VZV transcription and translation during latency are likely just as restricted as in HSV. Two reports showed that the protein detected in autopsy material is due to false-positive staining as a result of cross-reaction of VZV antibodies with blood group A1 antigens [35,36]. Furthermore, the presence of transcripts from several VZV ORFs in post-mortem material is likely due to early stages of VZV reactivation following death of the individuals since only ORF63 transcripts are detected when autopsy material is obtained less than 9 hours post-mortem [8]. Even more recently, targeted-enrichment for VZV transcripts, followed by next generation sequencing, detected ORF63 and a novel spliced transcript in 60% and 93%, respectively, of VZV-positive human TG analysed at short post-mortem interval [9]. This novel spliced transcript, termed “VZV latency-associated transcript” (VLT), is reminiscent of HSV LAT and antisense to ORF61, the homolog of HSV-1 ICP0. Both ORF61 and ICP0 are IE proteins that transactivate viral promoters required for lytic replication. Exogenous expression of ORF61 induces VZV reactivation in guinea pig enteric neurons [37]. However, ORF61 role during VZV reactivation is unknown and that of ICP0 during HSV-1 reactivation is controversial [38,39,40,41,42,43,44]. One hypothesis, based on exogenous expression of ORF61 and VLT, is that the latter represses ORF61 expression and activity, reducing reactivation [9]. However, there is no data yet on the function of VLT in the context of infection, including whether it plays a role in the establishment of latency or in the inhibition of reactivation. While much can be learned about the physical, steady state of latent VZV from autopsy material, there are shortcomings to these studies. First, death is a stressful process, rapidly inducing hypoxia in the ganglia where virus is latent, likely changing the molecular structure of viral genomes and transcription patterns. Second, mechanistic studies investigating viral and cellular factors required for establishment of, and reactivation from latency, are not currently possible with autopsy ganglia. Thus, a more fluid system, such as in vitro cultured human neurons, is required.

The following sections review the generation, characterization and use of in vitro cultured human neurons to study VZV latency and reactivation.

## 3. Generation and Characterization of Human Neurons to Study VZV

Human neuroblastoma SH-SY5Y cells [45] and rat DRG-mouse neuroblastoma ND7-23 cells [46] have been used to study VZV-neuron interaction and VZV latency and reactivation (reviewed in [47]). The use of such cell lines allows for large-scale cultures at a relatively low cost; however, these cells have limitations. ND7/23 cells were not terminally differentiated [46] and SH-SY5Y cells only differentiate to ~70% neuronal population. Efficient differentiation is imperative in the study of VZV latency, because non-neuronal cells efficiently replicate the virus, often leading to a lytic infection of the culture. Both cell lines are of neuroblastoma origin distinguishing them from neuronal identities, and the ND7-23 of murine origin. Together, these limitations make the interpretation of the results inherently and conceptually problematic. Importantly, VZV latency has not been established in either of these cell lines. More recently, VZV has been studied in human neurons derived from multipotent neural stem cells (hNSC) and pluripotent stem cells (PSCs) including human embryonic stem cells (hESC) and induced pluripotent stem cells (iPSC). Importantly, the nature of the precursor cell and the protocols used to differentiate neurons from hNSC, hESC or iPSC may differ, and may lead to the generation of different neuronal subtypes. A detailed description on the methods used to generate neurons from NSC and PSCs is beyond the scope of this review and reviewed elsewhere [48].

**Figure 1:**
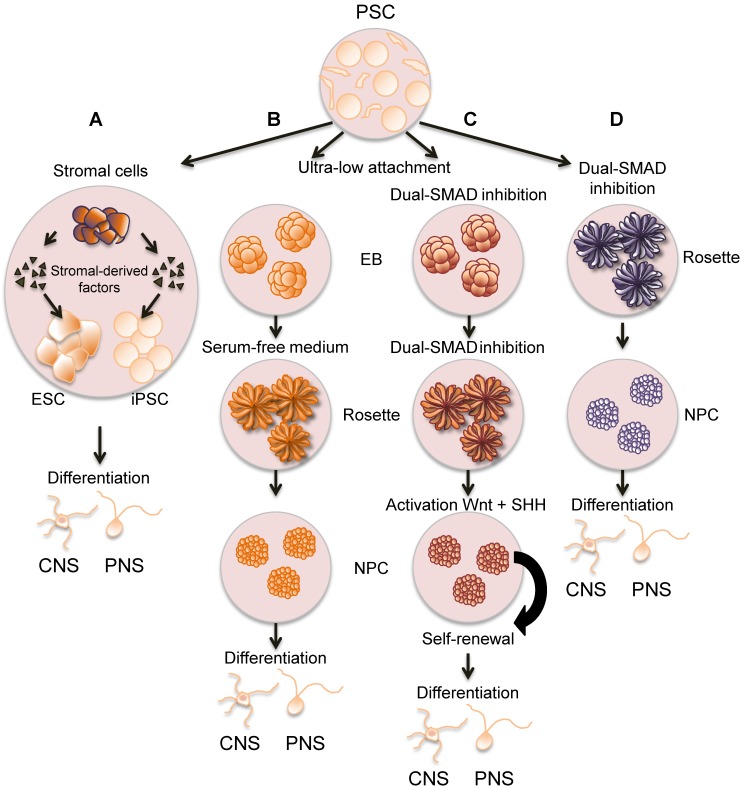
Derivation of human neurons from pluripotent stem cells (PSC). Schematic representation of the main procedures to generate human neurons from PSC, including embryonic stem cells (ESC) and induced pluripotent stem cells (iPSC). (**A**) Stromal cells release factors that facilitate the differentiation of PSCs into neurons resembling those from the central and peripheral nervous systems (CNS and PNS, respectively). (**B**) The use of ultra-low attachment conditions permits the formation of embryonic bodies (EB), which are grown in serum-free medium forming neural rosettes followed by neuronal precursor cells (NPC) that, upon differentiation, generate CNS- and PNS-like neurons. (**C**) A variation of this method includes the inhibition of dual-SMAD (small worm phenotype; mothers against decapentaplegic) and the activation of the Wnt (wingless and integrated) and sonic hedgehog (SHH) signaling pathways to facilitate self-renewal of the NPC, which are then differentiated as in (**B**). (**D**) A fast and efficient way of generating human neurons requires the inhibition of dual-SMAD without the formation of EB. In (**B**,**D**) the generated NPC cannot multiply.

In short, the main strategies to derive neurons from PSC include the use of stromal feeder co-culture systems, the formation of embryonic bodies (EBs), or the inhibition of dual-SMAD (*Caenorhabditis elegans* SMA, small worm phenotype; *Drosophila* MAD, mothers against decapentaplegic) with a cocktail of chemical compounds (Figure 1). SMADs are transcriptional coactivators or corepressors of the transforming growth factor (TGF-β) superfamily that plays very important roles during development and differentiation. In the first method, ESCs or iPSCs are co-cultured with stromal cell lines. This leads to direct differentiation of the stem cells into either central nervous system (CNS) or PNS neurons [49,50], depending on the source and combination of stromal cell lines and PSC. The most frequently used stromal cells are PA6 (derived from newborn calvaria tissue of C57BL/6 mice), MS5 (bone marrow cells derived from C3H/HeNSlc strain mice), S2 (*Drosophila melanogaster* cell line), and HepG2 (a human liver cancer cell line) [48]. The second method to differentiate PSCs into neurons requires their dissociation and culture in ultra-low attachment conditions facilitating the formation of three dimensional, sphere-like EBs [51]. Formation of EBs facilitates the natural and spontaneous differentiation towards cells of the three germ layers (ectoderm, mesoderm and endoderm). This resembles the differentiation process during the early embryogenesis stage. To prevent the differentiation of PSCs towards non-neural lineages present in mesoderm and endoderm, the EBs are cultured in serum-free medium, which favors the growth of NSC that can be further differentiated into neurons [52]. The third, and most efficient, method of differentiating PSCs into cells of the ectoderm lineage (without the generation of EBs) is called “dual-SMAD inhibition”. In this method, the SMAD-dependent TGF-β and bone morphogenic protein signaling pathways are inhibited by chemical compounds [53], leading to efficient neural conversion from PSCs with the intermediate generation of short-lived NSC. A limitation of this method is that the NSC cannot be efficiently expanded. More recently, a method that combines EB formation and dual-SMAD inhibition, plus simultaneous activation of both CNS and PNS patterning factors, has been developed. This procedure generates EBs enriched on self-renewable NSC, which can be switched to adherent culture conditions and further differentiated into specific neuronal subtypes [54]. The expandable NSC can be cryopreserved without losing proliferative and differentiation capabilities, providing a source for production of human neural cells with substantial reproducibility [54].

Just as there are many methods for in vitro neuronal differentiation, there are numerous types of neurons in vivo. VZV establishes latency in neurons of the PNS, making neurons with a peripheral fate ideal for in vitro models to study VZV latency and reactivation. However, there are different types of peripheral ganglia, each containing a heterogeneous population of neurons [55]. Determining the type of neurons being infected with VZV, and other alphaherpesviruses, is an area of current research. The PNS includes sensory (i.e., DRG and TG), autonomic (i.e., sympathetic and parasympathetic) and enteric branches. Both sensory and autonomic branches are innervated by CNS neurons originating from either the brain or spinal cord, establishing one functional connection throughout the whole nervous system. The enteric branch is not innervated by the CNS, thereby functioning independently.

The majority of sensory neurons can be functionally classified into 3 distinct populations: proprioceptive (sensing body position), mechanoreceptive (sensing touch, sound, pressure, stretch and others) and nociceptive (sensing noxious mechanical stimuli, chemicals, pain, temperature, and itch, among others). Generally, proprioceptive neurons have a large diameter and are heavily myelinated; mechanoreceptive neurons have a medium diameter with medium myelination, whereas nociceptive neurons have a small diameter with little-to-no myelination. The other major difference among neuronal subtypes is the specialized gene signature. Transcriptomic analysis of mouse DRG by single-cell RNA-Seq reveals there are 11 different subtypes of neurons with differential gene expression patterns [55,56]. Among the differentially expressed genes are transcription factors, neurofilaments, growth factor receptors, neuropeptides and ion channels. In humans, it is not clear whether VZV preferentially replicates or establishes latency in a specific neuronal subtype. Data from the SCID mouse model xenotransplanted with human DRG suggest that VZV replication is restricted at a post-entry event in large neurons (RT97^+^), but not in small neurons (Peripherin^+^) [57]. Therefore, it is important to characterize the neuronal population obtained upon differentiation since this may affect VZV replication.

In most cases of in vitro VZV infection, characterization of the neurons used is limited to the detection of a few pan-neuronal markers, such as β-III-tubulin or microtubule-associated protein 2 (MAP2); occasionally, specific markers of peripheral neurons (e.g., peripherin and Brn3a) are used. Examining such a limited number of markers may be misleading due to the overlap in gene expression among several subtypes of PNS neurons. If possible, several criteria, namely morphological parameters, expression patterns (including that of neurotransmitters) and functional properties, should be analyzed.

Despite having some deficits in the characterization of the derived neurons, several reports have provided highly relevant information on VZV infection of human neurons. One interesting observation is that infection of either iPSC- or NSC-derived human neurons with VZV at a very low multiplicity of infection (MOI, <0.001) does not result in cytopathic effect (CPE) during the first 2 weeks post-infection and viral DNA does not accumulate; however, infectious virus is produced and CPE is observed only at late time points post-infection (i.e., 4 weeks) [58,59,60,61]. Similarly, infection of human neurons forming three-dimensional tissue-like assemblies is productive without apparent CPE [62]. Conversely, infection of fibroblasts with the same low MOI leads to CPE in the first week [58,60]. Despite the differences in replication kinetics and pathogenicity, a transcriptomic analysis indicates that VZV transcription in fibroblasts and neurons is quite similar, suggesting VZV is not impaired in neurons [63]. Only 12 out of 71 VZV genes, inclusive of all kinetic classes, have minor (less than 2.5-fold) differences in expression between neurons and fibroblasts (ORFs 4, 8, 23, 28, 33.5, 36, 39, 50, 53, 54, 64/69, and 65) [63]. Infection of human DRG, hESC- and iPSC-derived neurons with a higher MOI leads to productive VZV infection [64,65,66,67].

Apart from examining VZV replication and transcription, human neurons have also been used to study VZV retrograde transport [66] and latency. The following section describes in vitro models to study VZV latency and reactivation.

## 4. In vitro Latency/Reactivation Models

The ideal in vitro model of alphaherpesvirus latency should fulfill the following criteria: (i) molecular characteristics recapitulate those seen in vivo: virus gene expression program is highly restricted (likely to one or two genes [68], VLT and ORF63 for VZV according to recent data [9]), very few, if any, viral mRNAs are translated, viral genomes are episomal and do not replicate, and infectious viral particles are not produced; (ii) latency is maintained for long periods of time in the majority of the infected neurons with rare spontaneous reactivation; and (iii) virus reactivates upon appropriate stimuli leading to production of infectious virus.

**Figure 2:**
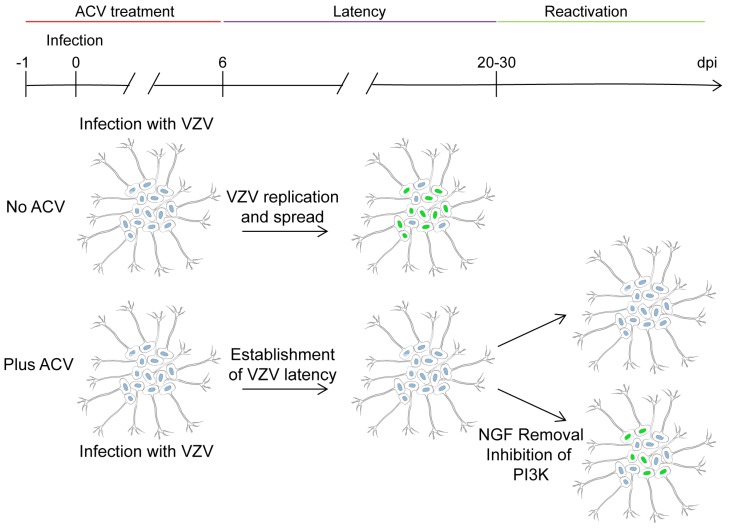
Schematic representation of the acyclovir (ACV) model to study VZV latency and reactivation (based on [74]). Addition of ACV to PSC-derived human neurons prior to infection followed by six days ACV incubation during VZV infection results in the establishment of a latent phenotype characterized by low level gene expression, no detectable protein expression and lack of infectious VZV during several weeks upon ACV removal. Infection is not abortive since VZV reactivates following removal of nerve growth factor (NGF) or inhibition of phosphoinositide 3-kinase (PI3K). In the original submission the authors used VZV expressing open reading frame 66 fused to green fluorescent protein, permitting the detection of infected neurons by direct fluorescence. Abbreviation: dpi, days post-infection.

These criteria for an in vitro latency model of alphaherpesviruses were first partially achieved by Wilcox and Johnson through infecting murine neurons with HSV-1 [69,70]. Briefly, rodent neurons cultured with acyclovir (ACV) were infected with HSV-1 in the presence of the drug for 6 days to terminate any replicating virus. ACV is a guanosine analogue that inhibits DNA replication by blocking elongation by the viral polymerase, but does not block viral transcription (with the exception of late genes which require viral DNA replication for expression). Treatment of neuronal cultures with ACV likely reduces the number of replicating viral genomes, thereby facilitating the suppression of viral transcription and entry into latency by cellular mechanisms. Importantly, ACV removal did not result in spontaneous HSV-1 reactivation (criterion ii) [69]. Wilcox and Johnson’s reports, as well as others, suggest reactivation of HSV-1 can be achieved by removing nerve growth factor (NGF) from culture medium or by inhibiting several enzymes including phosphoinositide 3-kinase (PI3K), Akt, or histone deacetylases (criterion iii) [69,70,71,72,73]. With this information in mind, Markus and colleagues established VZV-latency in vitro using two different models (Figure 2). Both models used hESC-derived human neurons (purity of differentiation was not directly examined in this study although previous publications from this group indicate that ~95% of the cells are neurons since they express β-III tubulin) infected with a recombinant cosmid-derived VZV (strain POka) in which the green fluorescent protein (GFP) coding sequence is inserted in-frame to the 5′ end of ORF66 coding sequence, resulting in the expression of a GFP-pORF66 fusion protein (POka-GFP66) [74]. In the first model, neurons were incubated with ACV for 24 h before low MOI (0.001) VZV infection and remained under ACV treatment for 6 days post-infection. In the absence of ACV, a productive, lytic infection is established with large numbers of neurons expressing GFP66; the number of GFP^+^ cells increases with time, suggesting virus replication and spread take place. On the contrary, ACV-treatment results in 50% of infected cultures to be GFP^−^. After ACV removal, all wells that were initially GFP^−^ remained GFP^−^ for up to 7 weeks after ACV removal, suggesting a lack of spontaneous reactivation. When cultures are incubated in the presence of ACV, VZV DNA is present at a low ratio of 2–3 copies/cell compared to 1000-fold more VZV DNA present in neurons infected without ACV. Whether VZV DNA is episomal in this model was not addressed. Following ACV treatment, transcriptome analysis reveals a VZV-gene expression profile nearly identical to that of non ACV-treated, productively infected cultures, but with a much lower magnitude of expression. Notably though, transcription of the genes flanking the unique short region is significantly enriched in ACV-treated, quiescent cultures compared to lytic ones. Despite gene transcription, GFP66 is not translated in ACV-treated cultures to levels detectable by fluorescence microscopy. Inhibition of PI3K or histone deacetylases, or removal of neurotrophic factors from the ACV-quiescent cultures, results in increased VZV genome copy number, transcription and protein expression. Interestingly, inhibition of PI3K in ACV-treated cultures incubated at 34 °C results in similar rates of reactivation as inhibition of histone deacetylases or removal of neurotropic factors, but the infection appears to spread and infectious virus is produced. This was suggested to be due to enhanced VZV replication at low temperature as previously shown in other cell types [75] and not to an effect of temperature on reactivation [74]. While this model provides interesting information, interpretation of the results should be taken cautiously due to the use of ACV. Because of its chain-terminator properties, any viral genome which incorporates ACV is likely “dead” and thus could never reactivate, leaving only genomes which entered latency before incorporation of ACV capable of reactivating at later times. It is currently unknown what differentiates genomes that begin replication and incorporate ACV versus genomes that go latent without replication, but understanding this mechanism could yield valuable information regarding the mechanism of how VZV establishes latency.

**Figure 3:**
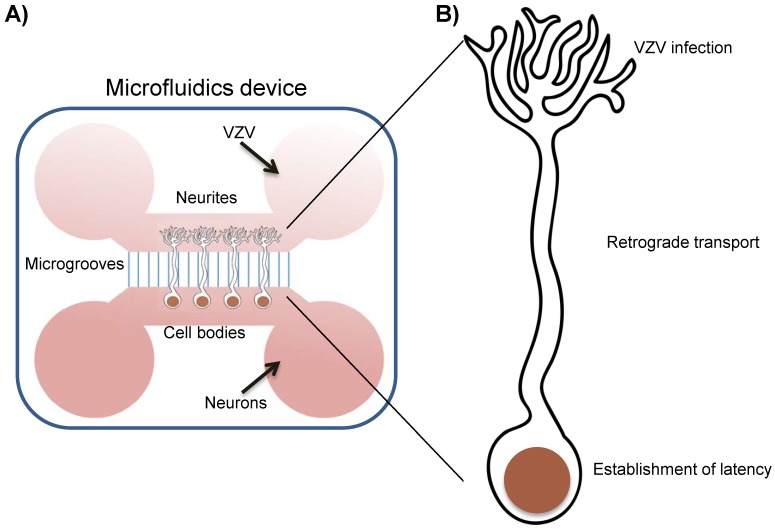
Establishment of VZV latency using microfluidic devices (based on [74,76]). (**A**) Cartoon showing a representation of a microfluidic device to separate the neuronal cell bodies from the neurites. Dissociated neurons are seeded in one of the chambers and growth factors are added in the opposite one inducing neurite outgrowth through the microgrooves. Before infection a pressure gradient is generated by increasing the volume of medium in the cell body compared to the neurite chamber. This ensures that VZV cannot diffuse in the medium to the cell body chamber when added to the neurite end. Therefore VZV can only reach the nucleus if it enters the neurite and is efficiently transported by cellular motors. (**B**) VZV infection through the neurite end leads to establishment of a latent-like phenotype that can be reactivated by inhibition of PI3K [74] or removal of NGF [76].

In the second model, Markus et al., cultured neurons in microfluidic devices, which physically separate somata and neurite termini, then infected them via the neurite end with a cell lysate of POka-GFP66-infected fibroblasts in the absence of ACV (Figure 3). This results in retrograde transport and delivery of viral genomes to the somata and low viral gene transcription without detection of GFP66 by microscopy. Following treatment with PI3K inhibitors, viral DNA and RNA levels increase at 37 °C; only when PI3K is inhibited at 34 °C is viral protein (GFP66) detected [74].

Sadaoka and colleagues [76] expanded on the compartmented, neurite infection model of Markus et al., to demonstrate the vaccine strain of VZV (VOka; Merck) is impaired for reactivation from latency compared to the POka strain. In this model, hESC-derived neurons were differentiated at >99% purity (β-III tubulin-positive staining). Following establishment of latency (without ACV), reactivation was initiated two weeks post-infection by removal of NGF from the culture medium and adding anti-NGF antibodies. Importantly, these authors demonstrated the VZV genome is in an episomal (or endless) configuration during latency, recapitulating what is known to occur in vivo [28]. However, all viral ORFs are transcribed at low levels during latency in both VOka- and POka-infected neurons.

Continuing the above model from Sadaoka, Kurapati and colleagues [77] further characterized the differentiated neurons as sensory based on expression of brain-specific homeobox/POU domain protein 3A (Brn3a), Islet1 and peripherin. The authors showed that c-Jun N-terminal kinase (JNK) is required for VZV replication and reactivation in neurons, as detected by transfer of infectious virus to non-neuronal cells. The role of JNK in VZV reactivation remains unknown, but it may be involved in a histone methyl/phospho switch, as it is for HSV-1 Phase I reactivation [73]. Studies using rat embryonic sympathetic neurons and ACV showed that HSV-1 reactivation occurs in two waves, termed Phase I, or animation, and Phase II [73]. Phase I is characterized by low-level genome-wide transcription in the absence of de novo protein synthesis and viral genome replication [73,78]. Conversely, Phase II is characterized by sequential IE, E and L kinetic class transcription, and requires both protein translation and viral DNA replication to progress completely through all three kinetic classes. Interestingly, HSV transcription during Phase I is initiated by histone methyl/phospho switches, dependent on JNK, which de-repress viral promoters even in the presence of methylated heterochromatic marks on histones [78]. The fact that stress pathways, including those induced by axotonomy, NGF deprivation, or nerve injury, induce signalling cascades that converge on JNK activation supports the role of this kinase in reactivation of alphaherpesviruses [71,79].

These models have many advantages; but, as discussed below, their transcription profiles do not reflect what has been found in human TG [9]. The reasons for this are currently unknown and require further investigation.

## 5. The Relevance of NGF in VZV Latency and Reactivation

NGF and its precursor protein, proNGF, signal through two receptors: TrkA and P75 (reviewed in [80]). Briefly, neuronal differentiation and survival are induced upon binding of NGF to TrkA [81,82]. Binding of pro-NGF to P75 induces apoptosis, whereas an NGF-P75 interaction has growth and survival effects. Moreover, P75 can interact with TrkA, thereby increasing the signalling activity of TrkA [83]. The specific role of TrkA, P75 or any other neurotrophic factor receptor in VZV latency has not been thoroughly characterized. Even so, inhibition of NGF signalling by removing NGF from culture medium induces reactivation in both the ACV-treated and compartmented models, suggesting a key role for this neurotrophin in VZV latency. This reflects the ex vivo situation in which impairment of NGF (or PI3K) signalling results in replication of VZV DNA in dissected post-mortem TG neurons; however, infectious virus is not recovered under these conditions [7].

Importantly, NGF signalling initiates numerous cascades, one of which includes PI3K, another molecule reported to be involved in VZV latency and reactivation (see above) [74]. However, Sadaoka and colleagues could not reactivate VZV using PI3K inhibitors, but did reactivate VZV when anti-NGF antibodies were employed [76], suggesting two distinct NGF signalling cascades may be involved in VZV latency. Whether the differences observed in reactivation between these two reports [74,76] are due to differences in the virus (cosmid-derived recombinant POka in Markus et al., versus non-recombinant POka in Sadaoka et al.), the neurons employed, or an unknown factor, is not understood at present. However, it seems that the differences in the neurons employed are less likely the cause of this discrepancy, since both groups used hESC-derived neurons showing similar percentages of neurons positive for β-III-tubulin, peripherin and Brn3a [74,76].

It is difficult to assess the number of neurons affected by blocking NGF signalling in VZV latency experiments since the expression level of NGF receptors and the percentage of neurons expressing them were not determined [74,76]. This may have an impact on the number of neurons in which VZV reactivates, potentially explaining why reactivation did not take place in all samples tested. Apart from NGF, other neurotrophic factors may be involved in maintaining VZV latency. Different neuronal subtypes express a distinct set of receptors for neurotrophic factors and this expression pattern changes during development and following birth. This makes it difficult to define the exact receptors and signalling pathways required to reactivate virus replication. For example, reactivation experiments using embryonic rat superior cervical ganglia latently infected with HSV-1 and inhibitors of both TrkA and P75 demonstrate continual signalling through TrkA, and not P75, is required to maintain HSV-1 latency [72]. In adult mouse sympathetic neurons; however, both NGF and glial cell-derived neurotrophic factor (GDNF) are important for maintaining HSV-1 latency, whereas they do not seem to play a role in adult TG neurons. In these sensory neurons, removal of neurturin and GDNF induces reactivation of HSV-1 and HSV-2, respectively [84].

Since VZV establishes latency in both sympathetic and sensory neurons, a thorough characterization of the neurons employed in the VZV latency models would help determine whether neurotrophic factors other than NGF are important for maintaining latency.

## 6. Concluding Remarks and Outlook

In the last few years, the field of VZV latency and reactivation has greatly advanced due to both the derivation of human neurons to perform mechanistic studies in vitro and advanced molecular techniques which led to the identification of VLT in vivo. The published VZV latency and reactivation models [74,76] fulfil most of the criteria mentioned above. While these models are quite complete and much can be learned from them, improvements to more closely replicate what occurs in vivo can still be aimed for. Areas to improve include, first, limiting the replication of viral genomes (average number of genomes increases with time in both studies); albeit such replication, if present, is insufficient to produce infectious virus. Second, the transcriptional profiles of the in vitro latently infected cultures closely align to productively infected ones [63], and do not match what is observed in human TG in which only VLT and ORF63 transcripts are detected [9,10]. This is similar to in vitro models of HSV-1 latency in which expression of genes from the three kinetic classes is detected [68,73], whereas in vivo, transcription is restricted to only the LAT region [9]. It is important to consider, for both HSV-1 and VZV, that detection of LAT or VLT and ORF63 transcripts, respectively, are from post-mortem ganglia, and the process of death may alter the virus-specific transcription profile in latently infected neurons. Importantly, the genome-wide gene expression of HSV or VZV during in vitro latency does not result in detectable levels of protein or production of infectious virus. However, there is data that low-level protein expression may occur in vivo, although not detectable by current methods, leading to T cell retention within ganglia (as reviewed in [85]).

Together, these details raise concerns as to whether the in vitro models represent a truly latent phenotype. There are several possible explanations for the presence of genome-wide expression during the latent-like phase in vitro. First, neurons were infected with a sonicated cell-free virus preparation which has contaminating mRNAs that can bind the neurons and remain adhered during the culturing, resulting in a lytic-like transcriptome profile. This is highly unlikely given the low stability of RNA. Second, the transcription observed in vitro may represent a continuum of Phase I-like reactivation events, possibly due to the short time in which latency is established in vitro compared to in vivo. This difference in time for latency establishment likely leads to less chromatinization of the viral genome with less stable, repressive epigenetic marks. Maintenance of latently infected neuronal cultures for longer periods may result in a gene expression profile more similar to the one observed in vivo. Third, the observed transcription corresponds to a low number of cells where lytic replication is occurring—either because latency was not established or because of spontaneous reactivation. These lytically infected cells may have enough transcripts for detection by sensitive techniques such as RT-qPCR or RNA-Seq, but do not result in efficient translation that can be detected by direct or indirect fluorescence. Finally, in vitro-derived neurons are not homogenous, containing cell types that may have different susceptibilities to VZV infection, establishment of latency and reactivation. The use of technologies investigating cellular processes at the single-cell level will aid in discerning this heterogeneity, and provide a better understanding of VZV latency and reactivation.

In the models above, numerous compounds are used (i.e., ACV for latency, PI3K or JNK inhibitors for reactivation). Because these compounds almost certainly have pleotropic effects, their inherent limitations must be kept in mind when interpreting the data, especially if future studies lacking such compounds yield different results.

Other possible ways to establish and maintain latency should also be explored. For example, both type 1 (α and β) and type 2 (γ) interferons (IFNs) inhibit virus replication. IFN-α, with or without ACV, induces latency of HSV-1 and PRV [86,87]; however, although antiviral, it is unable to induce VZV latency [88]. Similarly, IFN-γ inhibits transcription, replication and production of VZV in iPSC-derived human neurons, but it alone is insufficient to suppress virus into a latent state [60]. Both IFN-α and IFN-γ were used in “bulk” culture models of VZV infection, not in microfluidic chambers. It is possible that the cytokines would induce a latent state in these devices. While it has not been studied in terms of establishing latency, IFN-γ inhibits HSV-1 reactivation upon dissection of mouse TG harbouring latent virus [89]. Similar results were obtained when investigating reactivation from sympathetic rat neurons infected in vitro in the presence of ACV [90]. In this latter model, either IFN-β or IFN-γ was added at various time-points post-reactivation. Only when the cytokines are present during Phase I are they inhibitory; the presence of the IFN-inhibitor, ICP0, during Phase I blocks the inhibitory effect of IFN-γ. These results suggest that IFNs could be used to induce a VZV latent phenotype in human neurons or to inhibit reactivation.

While much has been learned about VZV latency and reactivation, there is still much more to discover. Only by understanding the mechanisms of this latency/lytic switch will we be able to restrict virus reactivation in vivo, and thus prevent zoster and the numerous other neurological manifestations of VZV reactivation.
